# Evolution of a Distinct SARS-CoV-2 Lineage Identified during an Investigation of a Hospital Outbreak

**DOI:** 10.3390/v16030337

**Published:** 2024-02-22

**Authors:** Hosoon Choi, Munok Hwang, Lisa Cornelius, Dhammika H. Navarathna, Piyali Chatterjee, Chetan Jinadatha

**Affiliations:** 1 Department of Research, Central Texas Veterans Health Care System, Temple, TX 76504, USA; munok.hwang@va.gov (M.H.); piyali.chatterjee@va.gov (P.C.); 2 Department of Medicine, Central Texas Veterans Health Care System, Temple, TX 76504, USA; lisa.cornelius@va.gov (L.C.); chetan.jinadatha@va.gov (C.J.); 3 Department of Pathology and Laboratory Medicine Services, Central Texas Veterans Health Care System, Temple, TX 76504, USA; dhammika.navarathna@va.gov; 4 School of Medicine, Texas A&M University, Bryan, TX 77807, USA

**Keywords:** SARS-CoV-2, evolution, outbreak

## Abstract

The SARS-CoV-2 virus steadily evolves, and numerous antigenically distinct variants have emerged over the past three years. Tracking the evolution of the virus would help us understand the process that generates the diverse variants and predict the future evolutionary trajectory of SARS-CoV-2. Here, we report the evolutionary trajectory of a unique Omicron lineage identified during an outbreak investigation that occurred in a residence unit in the healthcare system. The new lineage had four distinct non-synonymous and two distinct synonymous mutations apart from its parental lineage. Since this lineage of virus was exclusively found during the outbreak, we were able to track the detailed evolutionary history of the entire lineage along the transmission path. Furthermore, we estimated the evolutionary rate of the SARS-CoV-2 Omicron variant from the analysis of the evolution of the lineage. This new Omicron sub-lineage acquired 3 mutations in a 12-day period, and the evolutionary rate was estimated as 3.05 × 10^−3^ subs/site/year. This study provides more insight into an ever-evolving virus.

## 1. Introduction

Although the COVID-19 pandemic state of emergency is over, there is a need to envisage how the future of the SARS-CoV-2 virus could unfold. With access to an enormous amount of genomic data, we have been able to witness the evolutionary events of the virus in detail. SARS-CoV-2 viral genomes are unstable and continuously change over time, which results in the emergence of numerous variants [1]. Some variants of SARS-CoV-2 are antigenically distinct “quasispecies” of the virus that could breach the epidemiological barriers generated by previous SARS-CoV-2 infection and/or vaccination [2,3]. Tracking the evolution of the virus is a necessary step to lead us to understand the process that generates genomic diversity and would help us predict the future evolutionary trajectory of SARS-CoV-2.

Here, we report an analysis of the evolutionary path of SARS-CoV-2 using data from a distinct lineage. In an investigation of a hospital outbreak that occurred in a residential rehabilitation unit, we identified a potential new SARS-CoV-2 Omicron sub-lineage. We examined the mutations that occurred in this lineage along the well-defined transmission line and estimated the evolutionary rate of the Omicron sub-lineage. The purpose of this report is to provide insight into the understanding of the continued evolution of SARS-CoV-2 through the analysis of this rare event.

## 2. Methods

Nasopharyngeal swabs were collected from patients as part of routine COVID-19 screening during hospital admission. The SARS-CoV-2-positive samples identified by quantitative polymerase chain reaction (qPCR) were subjected to whole genome sequencing.

SARS-CoV-2 RNA was isolated using a QIAmp viral RNA mini kit (Qiagen, Hilden, Germany), and libraries for sequencing were prepared using a COVIDseq test kit (Illumina, San Diego, CA, USA), a next-generation sequencing test for detection of the SARS-CoV-2 virus with a set of tiling primers that have overlapping SARS-CoV-2 genome. The primer set was replaced with Artic v4 primers (https://github.com/artic-network/artic-ncov2019/tree/master/primer_schemes/nCoV-2019 (accessed on 2 January 2024), which is an improved primer set that reflects variations that occurred in the SARS-CoV-2 genome, especially for Omicron variants. The prepared libraries were sequenced in the NextSeq 550 system using the NextSeq 500/550 mid-output kit (Illumina) with paired-end reads.

The FASTQ files were generated using Local Run Manager software v2 and assembled through reference mapping with the SARS-CoV-2 reference genome (NC_045512) using the DRAGEN COVID lineage app at BaseSpace (Illumina). Lineage and mutations of the SARS-CoV-2 virus were analyzed using the Pangolin tool [4] and NextClade [5]. All sequencing was triplicated to rule out sequencing errors.

## 3. Results

### 3.1. A Potential New Omicron Sub-Lineage from Hospital Outbreak

**Figure 1:**
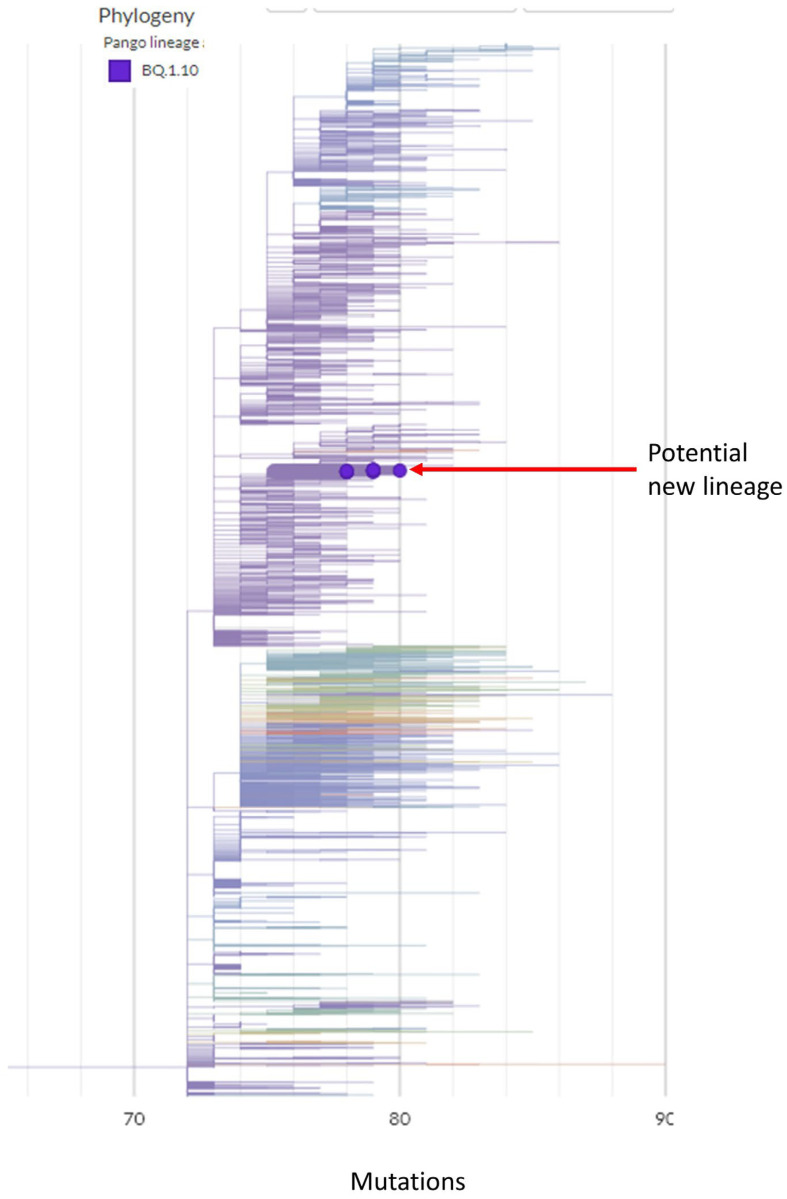
Phylogenetic tree of 13 potential new lineage samples. Relative position of potential new lineage amongst the global samples, obtained from Nextstrain (https://nextstrain.org/fetch/genome.ucsc.edu/trash/ct/subtreeAuspice1_genome_392fc_7cfc90.json?f_userOrOld=uploaded%20sample; accessed on 2 January 2024).

There was a total of 22 residents who were infected with SARS-CoV-2 as part of this outbreak investigation. A unique point of this outbreak is that a subset of patients (13 out of 22) was infected with a potential new SARS-CoV-2 lineage. The new variant was assigned as BQ.1.10. However, these 13 samples have distinct molecular characteristics in addition to the common mutations observed in BQ.1.10 (we arbitrarily named this sub-lineage BQ.1.10.DM to discriminate from other BQ.1.10). Only these 13 BQ.1.10.DM samples carry this characteristic mutational signature out of the over 10,000 SARS-CoV-2 samples sequenced in our lab. In addition, an intensive SARS-CoV-2 database search through over 6000 BQ.1.10 sequences from the Global Initiative on Sharing All Influenza Data, GISAID (www.gisaid.org; accessed on 25 September 2023) [6] had not identified any other BQ.1.10 sequences with the same distinct mutation as BQ.1.10.DM. The phylogenetic tree shows the relative position of BQ.1.10.DM sequences among the global samples (Figure 1). Because this lineage of virus is unique and was not identified afterward in any other location, we believe that these samples form a potential new lineage and that the beginning and end of this well-defined lineage of virus was contained in this outbreak event. The BQ.1.10.DM sub-lineage emerged six days after the outbreak and lasted till infection control intervened and ended the outbreak. BQ.1.10.DM could have emerged independently as other samples isolated from this outbreak event do not share any characteristic mutations that are observed in BQ.1.10.DM. In addition, since BQ.1.10.DM has not been identified in other regions, the regional factor might have affected its emergence.

The average Ct (cycle threshold) value of the BQ.1.10.DM samples obtained from qPCR was 23.1 which was lower than that of other lineage samples associated with the outbreak. The Ct value, which reports the number of cycles to pass the fluorescent signal threshold, indicates the amount of SARS-CoV-2 RNA in a patient’s specimen and is inversely proportional to the target material, i.e., SARS-CoV-2 RNA. The lower the Ct value, the more abundant target material in a sample. The amount of genetic material is an important factor in sequencing in order to get high-quality sequencing reads. Generally, it is necessary to have a Ct value of at most <30 to get an adequate amount of coverage [7]. The average sequencing coverage was 98.1% with 1876× read depth (Table 1). High sequencing coverage and depth are needed to determine the lineage of a virus. Generally, >90% sequencing coverage is necessary to be confident of the lineage call of a virus. As such, our sequencing coverage and depth is good enough for the analysis of genetic variations.

**Figure 2:**
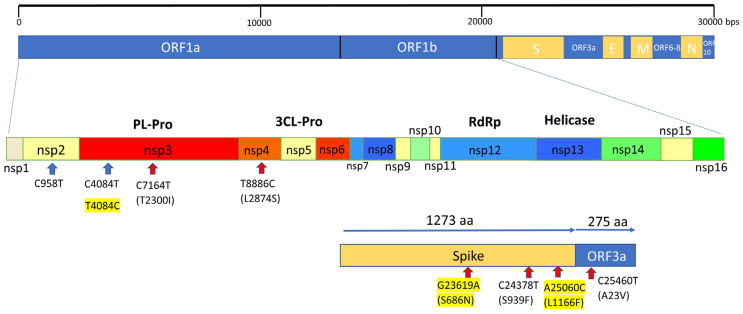
Position of mutations on the outbreak lineage. Synonymous mutations are marked with light blue arrows. Non-synonymous mutations are marked with red arrows. The three mutations that occurred during the outbreak are highlighted.

**Figure 3:**
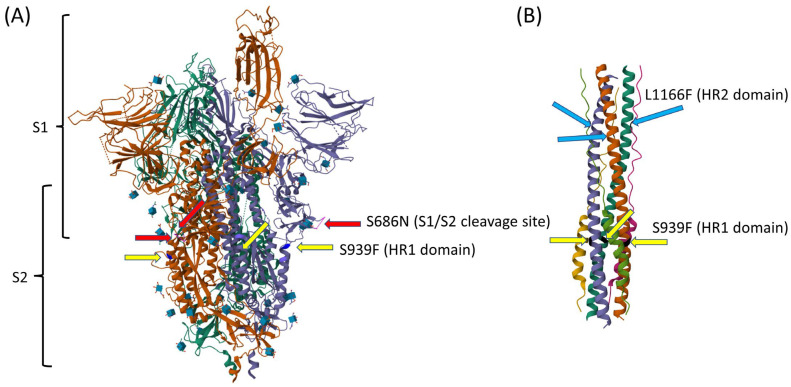
Cryo-EM structure of SARS-CoV-2 spike protein indicating the locations of mutations on each protomer. (**A**) Prefusion state of spike protein. HR2 domain is not included in the picture. (Adapted from RCSB PDB protein data bank; https://doi.org/10.2210/pdb7FCD/pdb [14]) (**B**) The folding of HR1 and HR2 of the S2 subunit during membrane fusion. Hydrophobic side chain of L1166 interacting with HR1. (Adapted from RCSB PDB protein data bank; https://doi.org/10.2210/pdb8CZI/pdb [15]).

As compared to the other reported BQ.1.10 on Pangolin, our 13 BQ.1.10.DM samples have 6 unique signature mutations (Table 1), 4 non-synonymous amino acid changes in the proteins, S: S939F, ORF1a: T2300I (nsp3), ORF1a: L2874S (nsp4), and ORF3a: A23V, and 2 synonymous mutations (Figure 2). The allele frequency of the mutation sites ranged from 92% to 100% which indicates that those altered alleles are predominant alleles. The mutation in spike protein S939F is in the HR1 domain and has been reported to affect immune response by relating to T-cell propensity [8] (Figure 3). Two mutations were observed in nonstructural proteins nsp3 and nsp4. The nsp3 papain-like protease (PL-pro) is a drug target for interrupting viral propagation [9]. The T2300I mutation is located on the well-conserved nsp4-binding Ecto domain region [10]. Nsp3 and nsp4 along with nsp6 form a double-membrane vesicle involved in viral replication [11,12]. ORF3a, an ion channel, is involved in virion release [13]. However, there are no reports on possible correlations between these mutations and phenotypes of the virus.

### 3.2. Evolutionary Path of a Potential New Omicron Sub-Lineage

**Figure 4:**
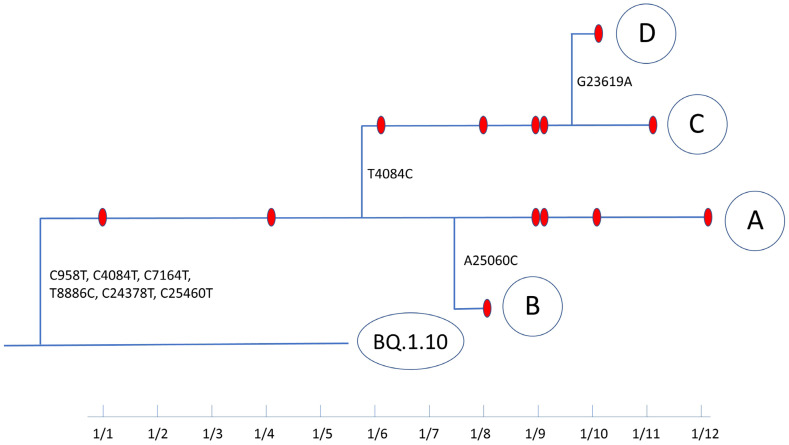
Mutations along the transmission path based on collection date and sequence changes. The sample IDs in Groups A–D are listed in Table 1.

We tracked the probable evolutionary path of BQ.1.10.DM over a 12-day period based on sequence changes and sample collection dates (Figure 4). In addition to the six characteristic mutations apart from the parental lineage, BQ.1.10.DM acquired three additional mutations in ORF1a and Spike protein along the transmission path. The 13 BQ.1.10.DM samples were divided into 4 groups, A–D based on the sequences. Within Group A, two branches formed: a mutation in Spike L1166F (A25060C) in the HR2 domain to form Group B and a reversion synonymous mutation from C4084T to T4084C to form Group C. Besides these two mutations, five viral transmissions occurred without any mutational changes. In Group C, a branch formed with an additional mutation in Spike S686N (G23619A) at the S1/S2 cleavage site to form Group D. Four transmission events occurred without mutation changes within this branch. There is no report or implication about the role of these two nonsynonymous mutations acquired by BQ.1.10.DM along its transmission path in virus properties. Since BQ.1.10.DM acquired 3 mutations during a 12-day period and the genome size of SARS-CoV-2 is 29.9 kb, the evolutionary rate of BQ.1.10.DM can be calculated as 3.05 × 10^−3^ subs/site/year.

Notably, out of nine mutations specific to BQ.1.10.DM (C958T, C4084T, C7164T, T8886C, C24378T, C25460T, A25060C, T4084C, and G23619A), five mutations are C-to-T mutations, which is postulated to occur as a result of accelerated APOBEC (apolipoprotein B mRNA editing enzyme)-mediated C-to-U deamination in the SARS-CoV-2 genome [16]. The APOBEC cytidine deaminases that play a role in controlling viral infections by inducing missense and nonsense mutations may also have a role in increasing the speed of mutations and facilitating the particular types of mutations in SARS-CoV-2 [17,18].

## 4. Discussion

Since the beginning of the pandemic in 2019, the SARS-CoV-2 virus has undergone numerous mutations and several variants of concern have emerged. Based on Pangolin, as of 25 September 2023, over 3370 lineages have been identified, and new variants such as BA.2.86 are still emerging [19,20].

Globally generated SARS-CoV-2 genome sequence data have revealed the constant emergence of mutations in the viral genome. During the early months of SARS-CoV-2 evolution, a single spike substitution (D614G), which conferred a growth advantage to the virus, arose and became dominant [21]. From the D614G progenitors, several SARS-CoV-2 variants have evolved and have been designated as variants of concern (VOCs) by the World Health Organization. The VOCs contain a number of non-synonymous mutations predominantly in the spike protein and show altered transmissibility and antigenicity. The spike protein is directly involved in cell entry. While several mutations (N679K, P681R, and P681H) enhance the furin-mediated cleavage of the S1-S2 site [22,23,24], most of the mutations are involved in antigenic escape [25]. As more and more humans are either infected or vaccinated, antigenic distance becomes a major factor in determining the fitness of the virus. There are limited studies of experimental characterization of the association between non-spike mutations and viral fitness. Mutations in the NSP6, N, M, and E proteins may modulate SARS-CoV-2 infectivity [26,27,28].

Most mutations are disadvantageous to the fitness of the virus. Each transmission event works as an evolutionary bottleneck during which most variations are lost, and non-functional viruses are selected against. Mutations that do not affect or are beneficial to viral fitness could accumulate over time. In the case of SARS-CoV-2, steadily increasing genetic diversity due to the enormous amount of viral genome replications in a substantial number of infected populations that occurred during the pandemic brought divergent and continuous evolution of the virus [1].

Understanding the evolution of SARS-CoV-2 outside of laboratory experiments is difficult. We utilized a rare opportunity to thoroughly examine the evolution of an entire well-defined lineage contained in an outbreak investigation and to obtain its evolutionary rate. Our evolutionary tracking of this distinct Omicron sub-lineage, conducted as part of an outbreak investigation demonstrated how the virus genome changes along the well-defined path of viral transmission.

Most of the mutations that were previously possessed or acquired during the course of the evolution of this lineage seem to have been passed on without selective advantage because the incidence rate of most characteristic mutations observed in BQ.1.10.DM was low and found only sporadically in several variants. If the mutation had a selective advantage, the occurrence of the mutation would have increased and been identified in many variants. There are no confirmed reports that show any selective advantage of the mutations found in the study. However, most of the observed non-synonymous mutations that occurred in the amino acids of the spike proteins (Figure 3), are involved in viral entry to the target cell. After S1/S2 cleavage, the HR1 and HR2 domains form a fusion core that is essential for membrane fusion and viral entry. These mutations occurred in the potentially important amino acids. Indeed, the occurrence rate of the spike protein mutation S939F in the HR1 domain has increased in the recently observed Omicron variants. In BA.2.86 and JN.1 variants, 99.09% of reported sequences have the S939F mutation. Therefore, the S939F mutation might have a selective advantage. The current relative growth advantage of variants with S939F mutation is calculated as 5% (https://cov-spectrum.org/explore/United%20States/AllSamples/AllTimes/variants?aaMutations=S%3AS939F&; accessed on 3 February 2024) by the fitness advantage calculation carried out by Chen et al. [29].

The trajectory of the BQ.1.10.DM demonstrated actual changes in the viral genome as the virus steadily diverged through its characteristic evolutionary rate until the lineage was contained by infection control. For investigating SARS-CoV-2 transmission, SARS-CoV-2 with 0 to 4 SNP differences were considered possibly related if there were plausible transmission events and they belonged to the same lineage [30]. This outbreak obviously showed the validity of this assumption. A total of three mutation events occurred in this clearly demarcated lineage of the virus during the outbreak transmission. Of note, out of 12 possible substitutions, more than half of the mutational events that occurred in this sub-lineage, BQ.1.10.DM, were C to T substitution mutations. This mutational bias of SARS-CoV-2 once more shown in this study is one of the main mechanisms to guide the trajectory and speed of the evolution of the virus.

The mutation rate of SARS-CoV-2, estimated as 1.3 × 10^−6^ ± 0.2 × 10^−6^ substitutions per base per infection cycle during in vitro propagation in Vero cells [31], is relatively low among RNA viruses [32] because of the proofreading mechanisms [33] unique to corona viruses. The viral mutation rate is an important factor in managing viral infections. For instance, the high mutation rate of HIV-1 enforced simultaneous multidrug treatment necessary for HIV-1 patients [34]. In other cases, the viral mutation rate was manipulated to inhibit viral reproduction. Molnupiravir, an antiviral medication for SARS-CoV-2 infection, inhibits viral reproduction by promoting viral RNA mutations to cause lethal mutagenesis [35,36]. The mutation rate is the basis of the evolutionary rate, which is a measure of accumulated mutations in a population in a given period of time. The mutation rate, population size, and generation times can all affect the evolutionary rate of a virus [37]. The viral evolutionary rate is one of the major factors that influence the emergence of variants of the virus [38], the viral fitness, and viral virulence [39,40] and also plays a role in vaccine effectiveness [41]. Estimating an accurate viral evolutionary rate is crucial to understanding the viral evolution and the emergence of antigenically distinct variants. Evolutionary rates of SARS-CoV-2 were obtained through cluster [42] or public database analysis [43]. In principle, getting the viral evolutionary rate is simple. Given the genome size of the virus and the time between the sample collection, the rate can be obtained by identifying the new mutations along with the transmission line. However, obtaining every mutation event in the entire lineage to calculate an accurate evolutionary rate is rarely feasible.

In early studies using sequence data obtained from the pre-Delta period, the evolutionary rate of SARS-CoV-2 was obtained as 2.3~4.6 × 10^−3^ subs/site/year [31,42,43] while Wang et al. estimated 0.67 × 10^−3^ subs/site/year [43]. It has been known that viral evolution gradually decreases over time due to selection pressure and the accumulation of harmful mutations [43] This study yielded an estimated evolutionary rate of 3.05 × 10^−3^ subs/site/year, which is consistent with previous studies performed during other variant surges. Our estimation indicates that the Omicron variants still have a high evolutionary rate.

This study clearly demonstrates how the virus genome changes along the well-defined path of viral transmission. The evolutionary rate of the Omicron sub-lineage was obtained from an analysis of a rare case in which we could track all the mutations in an entire lineage of the virus. In the development of vaccines and other control measures, it is important to consider the speed of viral adaptation to vaccines. The information from this study would provide an essential basis for the management of COVID-19 in an endemic era.

Limitations to our study include the fact that it was conducted at a single site and may not represent a global evolutionary rate as evolution among other subsegments of the population might be different. Additionally, although we included all the relevant samples we could obtain for the analysis, there is still a chance we may have missed some infections that evaded sample collection and sequencing. Using a single platform to perform sequencing could pose as another limitation. However, care was taken to minimize sequencing error by repeating the experiments in triplicate with high read depth and high sequence coverage.

## Figures and Tables

**Table 1:** Characteristics of the whole genome sequence of the potential new lineage.

Group	GISAID ID	Collection Date	Unique Mutations ^+,++^	Ct	Lineage	Sequence Coverage	Coverage Depth
A	hCoV-19/USA/TX-DM-9/2023	1 January 2023	C958T, C4084T, C7164T (ORF1a: T2300I) T8886C (ORF1a: L2874S), C24378T (S: S939F), C25460T (ORF3a: A23V)	28.5	BQ.1.10	96.97%	1619
	hCoV-19/USA/TX-DM-1/2023	4 January 2023		24.2	BQ.1.10	97.38%	2000
	hCoV-19/USA/TX-DM-8/2023	9 January 2023		27.4	BQ.1.10	98.02%	1713
	hCoV-19/USA/TX-DM-5/2023	9 January 2023		21.5	BQ.1.10	98.45%	1904
	hCoV-19/USA/TX-DM-11/2023	10 January 2023		29.6	BQ.1.10	97.06%	1546
	hCoV-19/USA/TX-DM-13/2023	12 January 2023		23.6	BQ.1.10	97.99%	1463
B	hCoV-19/USA/TX-DM-4/2023	8 January 2023	C958T, C4084T, C7164T (ORF1a: T2300I) T8886C (ORF1a: L2874S), C24378T (S: S939F), A25060C (S: L1166F), C25460T (ORF3a: A23V)	28.8	BQ.1.10	96.76%	1283
C	hCoV-19/USA/TX-DM-2/2023	16 January 2023	C958T, T4084C, C7164T (ORF1a: T2300I) T8886C (ORF1a: L2874S), C24378T (S: S939F), C25460T (ORF3a: A23V)	20.8	BQ.1.10	98.42%	2267
	hCoV-19/USA/TX-DM-3/2023	8 January 2023		18.2	BQ.1.10	98.89%	2075
	hCoV-19/USA/TX-DM-7/2023	9 January 2023		17.7	BQ.1.10	99.11%	2098
	hCoV-19/USA/TX-DM-6/2023	9 January 2023		21.5	BQ.1.10	98.80%	1890
	hCoV-19/USA/TX-DM-12/2023	11 January 2023		17.3	BQ.1.10	98.99%	2566
D	hCoV-19/USA/TX-DM-10/2023	10 January 2023	C958T, T4084C, C7164T (ORF1a: T2300I) T8886C (ORF1a: L2874S), G23619A (S: S686N), C24378T (S: S939F), C25460T (ORF3a: A23V)	22.8	BQ.1.10	98.50%	1962

Highlights indicate that an additional mutation occurred in each group along the path in Figure 4. ^+^ The presence of unique mutations was established by triplicate sequencing with high coverage. ^++^ Unique mutation other than the characteristic BQ.1.10 mutations (E:T9I M:D3N M:Q19E M:A63T N:P13L N:E136D N:R203K N:G204R N:S413R ORF1a:S135R ORF1a:Q556K ORF1a:T842I ORF1a:G1307S ORF1a:L3027F ORF1a:T3090I ORF1a:T3255I ORF1a:P3395H ORF1a:L3829F ORF1b:Y264H ORF1b:P314L ORF1b:M1156I ORF1b:R1315C ORF1b:I1566V ORF1b:T2163I ORF3a:T223I S:T19I S:A27S S:G142D S:V213G S:G339D S:S371F S:S373P S:S375F S:T376A S:D405N S:R408S S:K417N S:N440K S:K444T S:L452R S:N460K S:S477N S:T478K S:E484A S:F486V S:Q498R S:N501Y S:Y505H S:D614G S:H655Y S:N679K S:P681H S:N764K S:D796Y S:Q954H S:N969K; https://outbreak.info/situation-reports?xmin=2022-08-27&xmax=2023-02-27&pango=BQ.1.10; accessed on 31 March 2023).

## Data Availability

The sequences have been deposited in GenBank and the accession numbers are OR610672, OR610673, OR610674, OR610675, OR610676, OR610677, OR610678, OR610679, OR610680, OR610681, OR610682, OR610683, and OR610684.
